# Convolutional Neural Networks Enable Robust Automatic Segmentation of the Rat Hippocampus in MRI After Traumatic Brain Injury

**DOI:** 10.3389/fneur.2022.820267

**Published:** 2022-02-17

**Authors:** Riccardo De Feo, Elina Hämäläinen, Eppu Manninen, Riikka Immonen, Juan Miguel Valverde, Xavier Ekolle Ndode-Ekane, Olli Gröhn, Asla Pitkänen, Jussi Tohka

**Affiliations:** ^1^A. I. Virtanen Institute for Molecular Sciences, University of Eastern Finland, Kuopio, Finland; ^2^SAIMLAL Department (Human Anatomy, Histology, Forensic Medicine and Orthopedics), Sapienza Università di Roma, Rome, Italy

**Keywords:** brain MRI, CNN, deep learning, rat, registration, segmentation, TBI, U-net

## Abstract

Registration-based methods are commonly used in the automatic segmentation of magnetic resonance (MR) brain images. However, these methods are not robust to the presence of gross pathologies that can alter the brain anatomy and affect the alignment of the atlas image with the target image. In this work, we develop a robust algorithm, MU-Net-R, for automatic segmentation of the normal and injured rat hippocampus based on an ensemble of U-net-like Convolutional Neural Networks (CNNs). MU-Net-R was trained on manually segmented MR images of sham-operated rats and rats with traumatic brain injury (TBI) by lateral fluid percussion. The performance of MU-Net-R was quantitatively compared with methods based on single and multi-atlas registration using MR images from two large preclinical cohorts. Automatic segmentations using MU-Net-R and multi-atlas registration were of excellent quality, achieving cross-validated Dice scores above 0.90 despite the presence of brain lesions, atrophy, and ventricular enlargement. In contrast, the performance of single-atlas segmentation was unsatisfactory (cross-validated Dice scores below 0.85). Interestingly, the registration-based methods were better at segmenting the contralateral than the ipsilateral hippocampus, whereas MU-Net-R segmented the contralateral and ipsilateral hippocampus equally well. We assessed the progression of hippocampal damage after TBI by using our automatic segmentation tool. Our data show that the presence of TBI, time after TBI, and whether the hippocampus was ipsilateral or contralateral to the injury were the parameters that explained hippocampal volume.

## 1. Introduction

*In-vivo* magnetic resonance imaging (MRI) is a key technology for tracking neuroanatomical changes in brain diseases in both animal models and humans. Magnetic resonance imaging is non-invasive and allows longitudinal studies to be performed in living animals, with brains imaged in three dimensions (3D) at multiple time points. This enables monitoring of disease progression and therapeutic response in animal models of brain diseases. These models are vital in drug discovery and development of new treatments for brain diseases, in addition to their importance in basic research. Region segmentation (1, 2), which identifies specific brain regions of interest (ROIs) in 3D MRI scans, is an important component of many MRI data processing pipelines. In preclinical MRI, this step is often performed manually. However, manual segmentation is time consuming and suffers from considerable inter-rater and intra-rater variability (3).

A number of automated methods exist as alternatives to manual segmentation. Typically, these methods are based on registration, where a manually segmented template MR volume, termed atlas, is aligned to a target MR volume either via affine or deformable registration (4, 5). The segmentation of the template image can then be propagated to the target MR volume to be segmented using the found registration parameters. A single atlas coupled with a deformation model is usually insufficient to capture considerable anatomical variation (6). Therefore, registration-based segmentation is often improved by utilizing multiple atlases aligned to the same target volume and combining the resulting segmentation maps, an approach known as multi-atlas segmentation. The segmentation maps can be combined by majority voting or by more complex methods, such as Similarity and Truth Estimation for Propagated Segmentations (STEPS) (7). However, the final segmentation is still influenced by the registration quality. In addition, the heterogeneity introduced by brain diseases or traumatic brain injury (TBI) is harder to capture in a set of atlases. For example, depending on the impact force and its direction, TBI can result in large and multifocal lesions that significantly alter the brain anatomy with high interindividual variability (8). Consequently, aligning MRIs of the injured brain to the atlases can be challenging, especially when the ROIs are in the proximity of the primary brain lesion (9). While few methods have been proposed for the anatomical segmentation of brain MRIs with lesions on human data (10–13), the literature comparing the region segmentation accuracy of different methods in lesioned brains is limited and the potential biases arising from difference of the segmentation accuracy between healthy and lesioned brains has been rarely analyzed. In this regard (14) documented a significant drop in the performance of registration-based segmentation of the hippocampus due to atrophy, and (15) detected a drop in segmentation quality as a consequence of Huntington's disease across different segmentation methods.

For the segmentation of lesion brains, Convolutional Neural Networks (CNNs) (16) provide an interesting alternative to registration-based methods. Convolutional Neural Networks do not directly apply manually labeled atlases to the target anatomy, but use them to train the algorithm. Consequently, the information needed to segment a new image is encoded in the parameters of the neural network, eliminating the need for image registration. Convolutional Neural Networks have been successfully applied to a number of medical image segmentation tasks, sometimes achieving a segmentation accuracy comparable to that of human annotators. A prime example of CNNs in medical imaging is U-Net (17), based on an encoder-decoder architecture. U-Net inspired a large body of work such as its 3D adaptation V-Net (18), SEGNET (19), and DeepNAT (20), and has been combined with other architectural features such as attention (21) or Squeeze-and-Excitation blocks (22). Alternative approaches include work based on image-to-image translation (23) or mixed-scale architectures (24). For murine MRI, neural networks have been proposed for skull-stripping (25, 26), lesion segmentation (27, 28), and region segmentation (29). However, to our knowledge, CNNs have not been applied to the task of anatomical region segmentation of MRI of lesioned murine brains.

In this work, we develop a robust algorithm, MU-Net-R, for the automatic segmentation of the normal and injured rat hippocampus based on an ensemble of U-net-like CNNs. We further quantitatively compare MU-Net-R with state-of-the-art multi-atlas segmentation methods. We hypothesized that the performance of CNNs would not be affected by the presence of lesions to the same extent as in registration-based methods, as long as sufficient anatomical diversity of lesions was present in the training data. To ensure sufficient anatomical variability, we used MRI scans acquired at different time points after experimental TBI and obtained from two unusually large preclinical animal cohorts, EpiBioS4Rx (30, 31) and EPITARGET (32, 33). We focused on hippocampus segmentation because (a) it is frequently damaged by experimental and human TBI and (b) its damage has been associated with the development of posttraumatic epilepsy and cognitive impairment in both animal models and humans (34, 35). Because hippocampus segmentation is complicated by the presence of adjacent neocortical damage and atrophy, ventricular enlargements, and hippocampal distortions, it represents an ideal scenario to test our hypothesis. We additionally characterized the differences in segmentation quality between healthy and non-healthy anatomy, finding that the quality of segmentation maps generated by registration-based methods was reduced in the hemisphere ipsilateral to the lesion. We also show that CNNs reduce these differences and produce high quality segmentation maps under systematic visual inspection. Finally, we demonstrate an application of our method for assessing hippocampus volumes in a longitudinal study of TBI.

## 2. Materials and Methods

### 2.1. Materials

#### 2.1.1. EpiBioS4Rx Cohort

The Epilepsy Bioinformatics Study for Antiepileptogenic Therapy (EpiBioS4Rx, https://epibios.loni.usc.edu/) is an international multicenter study funded by National Institutes of Health with the goal of developing therapies to prevent posttraumatic epileptogenesis. The 7-month MRI follow-up of the EpiBioS4Rx animal cohort has been described in detail previously (30, 31). Here, we have analyzed the data from the University of Eastern Finland subcohort. We describe only the details that are important for the present study.

##### 2.1.1.1. Animals

Adult male Sprague-Dawley rats (Envigo Laboratories B.V., The Netherlands) were used. They were single-housed in a controlled environment (temperature 21–23°C, humidity 50–60%, lights on 7:00 a.m. to 7:00 p.m.) with free access to food and water. Severe TBI was induced in the left hemisphere by lateral fluid percussion under 4% isoflurane anesthesia (31). Sham-operated experimental controls underwent the same anesthesia and surgical procedures without the induction of the impact.

As summarized in Table 1, the entire cohort included 56 rats (13 sham and 43 with TBI), of which the 12 (5 sham, 7 TBI) first animals to complete follow-up were selected for manual annotation of the hippocampus. Mean impact pressure was 2.87 ± 0.82 atm in the entire cohort and 2.92 ± 1.37 atm in the manual annotation subcohort.

**Table 1:** Number of MRI scans per cohort (EpiBioS4Rx, EPITARGET) in different time points and treatment groups [TBI, sham-operated experimental controls].

**Timepoint**	**TBI**	**Sham**
**EpiBioS4Rx**		
2 d	43 (7,0)	12 (5,1)
9 d	42 (6,1)	13 (5,1)
30 d	42 (6,1)	13 (5,0)
150 d	40 (7,1)	13 (5,1)
**EPITARGET**		
2 d	118 (4,0)	23 (2,2)
7 d	117 (4,2)	23 (2,0)
21 d	117 (4,0)	23 (2,2)

*In parenthesis, the first number indicates the number of volumes used for manual annotation of the hippocampus and the second indicates the number of volumes used for manual annotation of brain masks. d, day; TBI, traumatic brain injury*.

##### 2.1.1.2. MRI

Rats were imaged 2 days (d), 9 d, 1 month, and 5 months after TBI or sham surgery (Table 1) using a 7-Tesla Bruker PharmaScan MRI scanner (Bruker BioSpin MRI GmbH, Ettlingen, Germany). During imaging, rats were anesthetized with isoflurane. A volume coil was used as radiofrequency transmitter and a quadrature surface coil designed for the rat brain was used as receiver. Local magnetic field inhomogeneity was minimized using a three-dimensional field map-based shimming protocol. All images were acquired using a three-dimensional multi-gradient echo sequence. A train of 13 echoes was acquired, where the first echo time was 2.7 ms, the echo time separation was 3.1 ms, and the last echo time was 39.9 ms. The voxel size was 0.16 × 0.16 × 0.16 mm^3^, the repetition time was 66 ms, the flip angle was 16, the number of signal averages was 1, and the imaging time was 10 min 44 s. Images with different echo times were summed to produce a high signal-to-noise ratio image for segmentation and image registration.

#### 2.1.2. EPITARGET Cohort

EPITARGET (https://epitarget.eu/) was a European Union Framework 7—funded, large-scale, multidisciplinary research project aimed at identifying mechanisms and treatment targets for epileptogenesis after various epileptogenic brain insults. The 6-month MRI follow-up of the EPITARGET animal cohort has been described in detail previously (32, 33). We describe only the details that are important for the present study.

##### 2.1.2.1. Animals

Adult male Sprague-Dawley rats (Envigo Laboratories S.r.l., Udine, Italy) were used for the study. The housing and induction of left hemisphere TBI or sham injury were as described for the EpiBioS4Rx cohort. However, injury surgery was performed under pentobarbital-based anesthesia instead of isoflurane. The entire cohort included 144 rats, and images from the first six rats (two sham, four TBI) were selected for manual annotation of the hippocampus. Mean impact pressure was 3.26 ± 0.08 atm in the entire cohort and 3.22 ± 0.02 atm in the manual annotation subcohort.

##### 2.1.2.2. MRI

Imaging was performed as described for the EpiBioS4Rx cohort, except that (a) imaging was performed 2, 7, and 21 days after TBI or sham surgery (Table 1) and (b) all images were acquired with a two-dimensional multislice multigradient echo sequence. A train of 12 echoes was collected, where the first echo time was 4 ms, the echo time separation was 5 ms, and the last echo time was 59 ms. In-plane image resolution was 0.15 × 0.15 mm^2^, slice thickness was 0.5 mm, number of slices was 24, repetition time was 1.643 s, flip angle was 45°, number of signal averages was 4, and imaging time was 11 min 37 s. Images with different echo times were summed to produce a high signal-to-noise ratio image for segmentation and image registration.

### 2.2. Manual Annotation

For outlining the ROIs, the 3D (EpiBioS4Rx) and multi-slice 2D (EPITARGET) ${\text{T}}_{2}^{*}$-weighted MRI images were imported as NIfTI files (.nii) into Aedes 1.0 (http://aedes.uef.fi) - an in-house tool with graphical user interface for medical image analysis. Aedes is available at http://aedes.uef.fi/ and runs under MATLAB (MATLAB Release 2018b, The MathWorks, Inc.).

#### 2.2.1. Manual Segmentation of the Brain Mask

A trained researcher (E.H.) outlined the brain surface on 160 μ*m*-thick (EpiBioS4Rx) or 150 μ*m*-thick (EPITARGET) horizontal MRI slices, covering the entire dorsoventral extent of the cerebrum (excluding the olfactory bulbs and cerebellum). In addition, E.H. outlined the brain surface on 160 μ*m*-thick (EpiBioS4Rx) or 500 μ*m*-thick (EPITARGET) coronal brain slices to increase the accuracy of dorsal and ventral delineation of the brain surface (Supplementary Figures 1, 2). In the EpiBioS4Rx cohort, we drew the whole brain outline for 6 scans from 6 different rats, outlining on average 33.7 ± 1.4 (range 31 – 37) horizontal slices for each MRI scan. In the EPITARGET cohort, we prepared the whole brain mask for six rats and the mean number of MRI slices outlined per case was 10.8 ± 0.9 (range 10–12).

##### 2.2.1.1. Manual Segmentation of the Hippocampus

Outlines of the ipsilateral (left) and contralateral hippocampus were drawn by E.H. on each coronal MRI slice where the hippocampus was present (slice thickness in EpiBioS4Rx 0.16 mm and in EPITARGET 0.50 mm). In addition to the hippocampus proper and the dentate gyrus, the outlines included the fimbria fornix, but excluded the subiculum (Supplementary Figures 3, 4). Manual annotation was performed with the help of thionin-stained coronal 30 μ*m*-thick histological sections of the same brain available at the end of follow-up, and with the Paxinos rat brain atlas (36). In the EpiBioS4Rx cohort, we outlined the hippocampi of 15 rats (8 TBI, 7 sham) imaged at 2, 9, 30 days, and/or 5 months post-injury or sham surgery. In the EPITARGET cohort, we outlined the hippocampi of six rats (four TBI, two sham) imaged 2, 7, and/or 21 days after TBI or sham surgery.

##### 2.2.1.2. Brain Mask Completion

As described above, only six brain masks were manually labeled in the EpiBioS4Rx dataset and every second sagittal slice was annotated. To reconstruct complete brain masks, we first applied a binary closing operation with a hand-crafted kernel to reconstruct the brain mask, and then filled any remaining hole in the mask volume (Supplementary Figure 5). Morphological operations were implemented using the scikit-image library (37).

To generate brain masks for the complete training datasets, we trained a single 3D CNN for each dataset as described in Section 2.3, using the same overall structure and number of channels, but limiting the output to the brain mask. Using this network, we generated brain-mask labels for the remaining animals, so that our CNN could be trained on this data for both skull stripping and hippocampus segmentation.

### 2.3. CNN-Based Segmentation

#### 2.3.1. CNN Architecture

**Figure 1:**
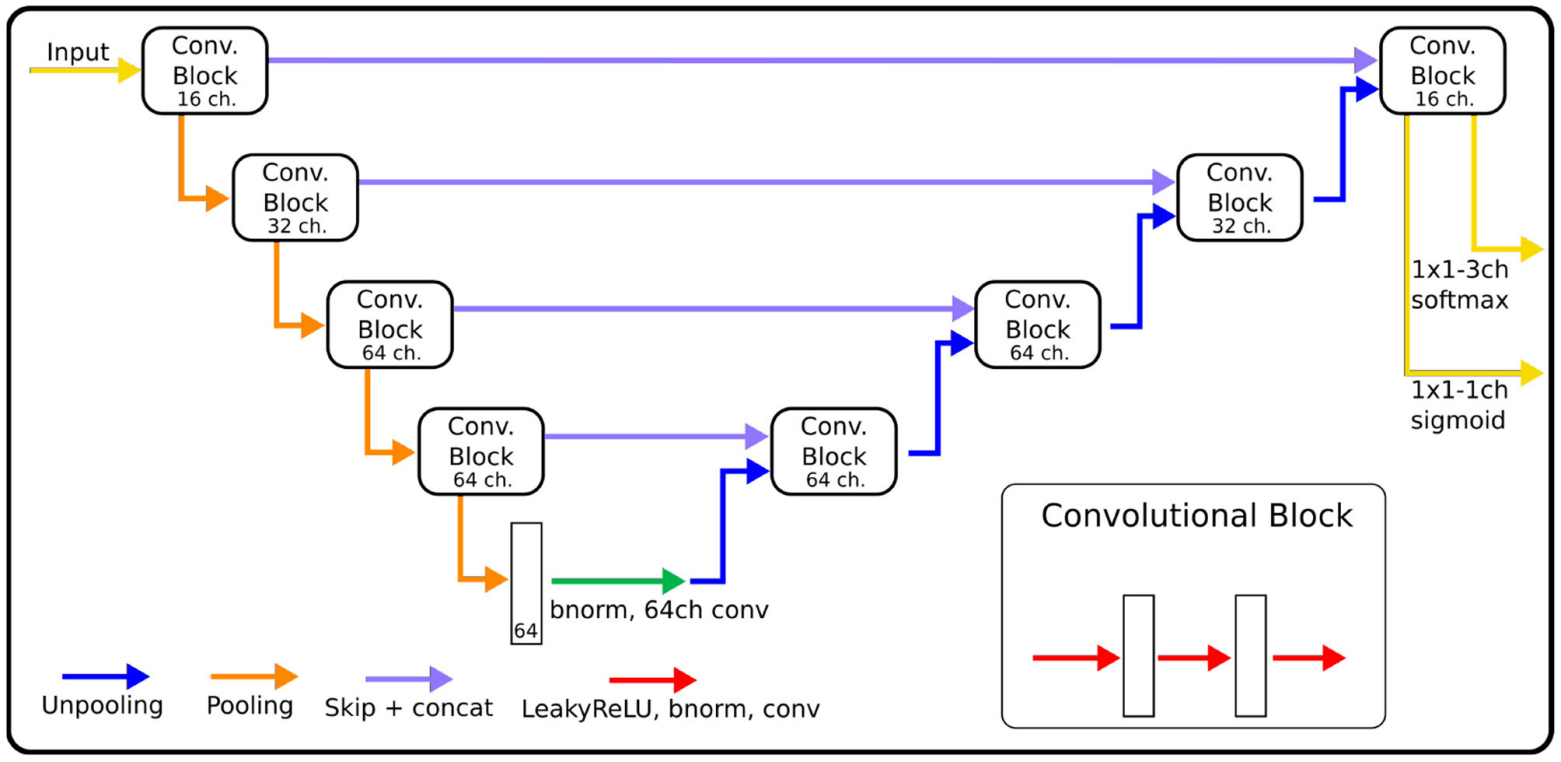
The architecture of CNNs, indicating for each block the number of channels used in each convolution. The size of convolution kernels is 3×3×3 for the EpiBioS4Rx data, and 3×3 for the EPITARGET data.

The architecture of our CNN (Figure 1) is based on MU-Net (29), which in turn was inspired by U-Net (17) and DeepNAT (20), to perform simultaneous region segmentation and skull stripping of mouse brain MRI. Our network exhibits a U-Net-like encoder/decoder structure, where the encoder and decoder branches are connected by a bottleneck layer and by skip connections between corresponding encoder and decoder stages. Pooling operations connect shallower blocks on the encoder branch to the deeper blocks, halving the size of the feature maps, while unpooling layers (38) connect different decoder blocks to the higher resolution ones, reversing the pooling operation.

Each block consists of three iterations of Leaky ReLU activation (39), batch normalization (40) and convolution. From the shallowest to the deepest convolution block, each convolution within the block uses 16, 32, 64, and 64 channels, respectively (Figure 1). Throughout the rest of this paper, we will refer to the network we trained for rat hippocampus segmentation as MU-Net-R.

We opted for a different choice regarding the dimension of the filters for each dataset. Since the EPITARGET ${\text{T}}_{2}^{*}$ MRI data are highly anisotropic, with higher resolution on coronal slices than in the fronto-caudal direction, we used 2D filters (3 × 3) in the coronal plane. This choice was based on our previous work (29) as well as (41), which indicated that a 2D convolutions were preferable for the segmentation of images with anisotropic voxel size. Conversely, in the network trained on isotropic EpiBioS4Rx ${\text{T}}_{2}^{*}$ data, we preferred 3D filters (3 × 3 × 3).

The architecture here described differs from MU-Net in the number of convolution operations, as MU-Net always employs 64-channels convolutions, and in the filter size, whereas MU-Net utilized 5x5 convolutions. These modifications reduce the total number of parameters from 2,087,944 (2D) and 10,286,344 (3D) to, respectively, 428,436 and 1,125,716. In this way we achieved the same segmentation quality as MU-Net with a lower number of parameters. The comparison with MU-Net and with a single network instead of ensembling is displayed in the ablation studies outlined in the Supplementary Table 1.

#### 2.3.2. Loss Function

The loss function is an evaluation criterion minimized during the optimization of the network. Our loss function is composed of two terms, referring to the skull-stripping task (*L*_*Brain*_) and the hippocampus segmentation task (*L*_*HC*_):


(1) $$\begin{array}{l} L=L_{HC}+L_{Brain}. \end{array}$$

Let the prediction for label *l* at voxel *n* be defined as *p*_*ln*_, and the corresponding ground-truth value as *y*_*ln*_. For the anatomical segmentation of the hippocampus, a task including three classes (ipsilateral hippocampus, contralateral hippocampus, and background), we employed the generalized Dice loss (42) written as:


(2) $$\begin{array}{l} L_{HC}=1-2\frac{\sum_{l=1}^{3}w_{l}\sum_{n}y_{ln}p_{ln}}{\sum_{l=1}^{3}w_{l}\sum_{n}y_{ln}+p_{ln}}, \end{array}$$

with the weight parameters *w*_*l*_ defined as $w_{l}={\left(\sum p_{ln}\right)}^{-2}$ following (42). In practice, the weight parameters favor the hippocampus classes over the large background class. Maintaining the naming conventions, for the skull-stripping task we used the following Dice loss term:


(3) $$\begin{array}{l} L_{Brain}=-\frac{\sum_{n}y_{n}p_{n}}{\sum_{n}y_{n}+p_{n}}. \end{array}$$

#### 2.3.3. Training

We minimized *L* with stochastic gradient descent using the RAdam optimizer (43). RAdam is an optimizer based on Adam (44) designed to better avoid local optima, obtain more generalizable neural networks, and train in fewer epochs. We utilized RAdam with default parameters (learning rate of 0.001, β_1_ = 0.9, β_2_ = 0.999 and no weight decay) and a batch size of one, as constrained by the available GPU memory. Networks and training were implemented in PyTorch and ran on a workstation with a GeForce RTX 2080 Ti GPU, 64 GB RAM and an AMD Ryzen 9 3900X 12- Core Processor. MU-Net-R networks were trained for up to 250 epochs, or until the average validation loss did not improve during the last 10 epochs.

During training, data were augmented online: each time an image was loaded, we randomly applied with a 50% probability a scaling transformation by a factor of α, randomly drawn from the interval [0.95, 1.05]. Each MRI volume was independently normalized to have a mean of zero and unit variance. To avoid interpolation issues with the low-resolution data, and taking into account that while biological variability is important the animal's orientation is strictly controlled during data acquisition, we did not include rotations in our data augmentation scheme.

#### 2.3.4. Post-processing

We applied a simple post-processing procedure to each CNN-generated segmentation. We selected the largest connected component of the MU-Net-R segmentation to represent each segmented region (brain mask and hippocampus) using the label function from scikit-image (37). We then filled all holes in this component using binary_fill_holes from scipy.ndimage (45).

### 2.4. Registration-Based Segmentation

#### 2.4.1. Registration

We compared CNN-based segmentation maps with single and multi-atlas-based segmentation maps. To do this, we performed image registration using Advanced Normalization Tools (46) to facilitate the transfer of manual segmentations of the hippocampus to other brains with single- and multi-atlas approaches. Before image registration, the images were skull-stripped using FMRIB Software Library's Brain Extraction Tool [FSL BET (47)]. We selected FSL BET for this step instead of using the masks generated by the CNN to keep the two pipelines completely independent, and ensure registration-based methods would be representative of typical registration-based results used in preclinical research. The masked images were then used for image registration. The brain masks for the EPITARGET dataset computed using FSL BET included marked amounts of non-brain tissue associated with the experimental TBI, which resulted in inaccurate image registrations. To improve the brain masks, we first registered the images to one of the brain images using rigid-body and affine transformations. The FSL BET brain mask of that image was then manually refined and transformed to the rest of the brain images, resulting in more accurate brain masks and registrations.

Image registration between a template brain and a target brain volume included the computation of a rigid-body transformation, an affine transformation, and a Symmetric image Normalization (SyN) transformation. We used global correlation as the similarity metric for the rigid-body and affine transformations and neighborhood cross-correlation for the SyN transformation. The computed transforms were then applied to the template brain's sum-over-echoes ${\text{T}}_{2}^{*}$-weighted image as well as its manually labeled hippocampi. All operations described in this section were performed on a 6-core AMD Ryzen 5 5600X processor.

#### 2.4.2. Single-Atlas Segmentation

For each template registered to a target brain, we applied the same transforms to the label map of the template, using nearest neighbor interpolation. Each measure reported for single-atlas segmentation in this work was an average between that of each individual single-atlas segmentation map for the same target brain.

#### 2.4.3. Multi-Atlas Segmentation

To label the hippocampus in each target volume by combining the individually-registered atlases we applied two different label fusion strategies: STEPS multi-atlas segmentation (48) and majority voting. Similarity and truth estimation for propagated segmentation combines multiple registered label maps by taking into account the local and global matching between the deformed templates and the target MRI volume. It does so by utilizing at the same time an expectation-maximization approach and Markov Random Fields to improve the segmentation based on the quality of the registration. We applied STEPS implementation distributed with NiftySeg (7, 48).

Similarity and truth estimation for propagated segmentation depends on two variables: the standard deviation of its Gaussian kernel and the number of volumes employed. Given the limited size of our dataset, and to reduce the risk of overfitting, we chose the standard deviation found in our previous work (29) as a result of a grid search between volumes aligned using diffeomorphic registration. When labeling each volume, we used all available registered atlases in label fusion for both STEPS and majority voting. The majority voting refers to choosing the most commonly occurring label among all registered atlases for each voxel.

For all atlas-based methods, i.e. single-atlas, STEPS, and majority voting, we only used as atlases those scans acquired at the same time-point.

### 2.5. Evaluation Metrics

We compared the segmentation maps to the manually annotated ground truth using the following metrics: Dice score, Hausdorff 95 distance, volume similarity (VS), compactness score (CS), precision, and recall.

#### 2.5.1. Dice Score

The Dice score (49) is a measure of the overlap between two volumes and is defined as:


(4) $$\begin{array}{l} D=\frac{2|Y_{t}\cap Y|}{\left|Y_{t}|+|Y\right|}, \end{array}$$

where *Y* is the automatic segmentation and *Y*_*t*_ the ground truth. A score of 1 corresponds to a perfect overlap and a score of 0 to a complete absence of overlap.

##### 2.5.1.1. Hausdorff 95

The Hausdorff distance (HD) (50) refers to the magnitude of the largest segmentation error of the prediction when compared to the ground truth:


(5) $$\begin{array}{l} HD\left(Y,Y_{t}\right)=\max\left(h\left(Y,Y_{t}\right),h\left(Y_{t},Y\right)\right) \end{array}$$

where


(6) $$\begin{array}{l} h\left(Y,Y_{t}\right)=\underset{y\in Y}{\max}\underset{y_{t}\in Y_{t}}{\min}|y-y_{t}|. \end{array}$$

We evaluated the 95th percentile of the Hausdorff distance, denoted as HD95, and measured it in millimeters. HD95 was calculated using MedPy (51).

##### 2.5.1.2. Volume Similarity

We measured the VS between prediction and ground truth, following the definition provided by (52). Unlike the Dice score, VS does not depend on the overlap between the two regions, and only depends on their volumes:


(7) $$\begin{array}{l} VS=1-\frac{\left||Y_{t}|-|Y|\right|}{\left|Y_{t}|+|Y\right|}. \end{array}$$

##### 2.5.1.3. Compactness Score

Compactness is defined as the ratio between area and volume (53):


(8) $$\begin{array}{l} Compactness=area^{1.5}/volume. \end{array}$$

We define a CS to indicate how close the compactness of the ROI segmentation mask *C* is to the compactness of the ground truth *C*_*GT*_. The CS is defined:


(9) $$\begin{array}{l} CS=1-2\frac{C-C_{GT}}{C+C_{GT}}, \end{array}$$

where *CS* = 1 indicates an identical compactness, and lower values indicate the two regions display a different ratio between surface and volume. To calculate the compactness, we used code from (27).

##### 2.5.1.4. Precision and Recall

Precision *P* and recall *R* evaluate, respectively, the ratio between true positives and the total number of positive predictions, and true positives and ground truth size. As such, increasing the number of false positives reduces the precision, and increasing the number of false negatives reduces recall. Both metrics vary between 0 and 1, and are defined as:


(10) $$\begin{array}{l} P=\frac{\left|Y_{t}\cap Y\right|}{\left|Y\right|},\;R=\frac{\left|Y_{t}\cap Y\right|}{\left|Y_{t}\right|}. \end{array}$$

### 2.6. Cross-Validation

We used cross-validation to evaluate CNN-based segmentation maps as well as the registration-based segmentation maps. For both the EpiBioS4Rx and the EPITARGET datasets, we applied six-fold cross-validation. The labeled samples from the EpiBioS4Rx dataset were divided into six-folds, where each fold contained two animals. Likewise, for the EPITARGET dataset, we defined six-folds, each containing one animal.

#### 2.6.1. Registration

For each test fold, the remaining data was utilized as atlases to label the test fold according to the different methods outlined in Section 2.4. Registrations were performed within each time point. Thus for EpiBioS4Rx the brain of each of the 12 animals was registered to 10 other brains at each of the four time points, which would have resulted in 480 image registrations. However, labeled images for one brain at two time points were missing in the EpiBioS4Rx dataset (Table 1), reducing the total to 440 image registrations. For the EPITARGET dataset, the brain of each of the six animals was registered to the five other brains at each of the three time points, resulting in 90 image registrations.

#### 2.6.2. MU-Net-R

For MU-Net-R the results were evaluated by selecting each fold as the testing data of one ensemble of networks, trained using the remaining data. To train each ensemble with early stopping we applied nested six-fold cross validation: the training set was further randomly divided into six-folds, using one fold as validation data during the training loop. In this way, we trained an ensemble of six networks, one for each validation fold. The final prediction for the test fold was the majority voting prediction from all networks generated from the same training set.

#### 2.6.3. Tests of Statistical Significance

We evaluated the differences in average values of evaluation metrics between different segmentation methods utilizing the paired permutation test (54) implemented using the permute library (https://statlab.github.io/permute/). We used 10,000 iterations for the permutation tests and applied Bonferroni's correction for multiple comparisons.

### 2.7. Visual Evaluation

After segmenting all hippocampi from the EpiBioS4Rx dataset (56 animals across four timepoints), our annotator (E.H.) evaluated every segmented volume according to the following procedure: For each volume, one coronal slice at a time was selected, starting caudally, and proceeding in the rostral direction. For each slice, the annotator was asked to input four numbers, corresponding to an evaluation for the dorsal and ventral parts of the left and right hippocampus. The evaluation scale, inspired by (55), was as follows:

1. Acceptable “as is”
2. Minor differences. Minor edits necessary. A small number of voxels, or <20% of the area
3. Moderate edits required, 20–50% of the area would need to be changed
4. Major edits required, >50% of the area would need to be manually edited
5. Gross error, no resemblance to the anatomical structure.

We simultaneously displayed the unlabeled MRI slice side-by-side with the same MRI slice overlaid with the ipsilateral hippocampus highlighted in red, and the contralateral one in blue. The volumes were presented to the annotator in a randomized order.

In addition to every labeled slice, we evaluated two additional slices in each direction, rostrally and caudally, to allow for the detection of hippocampal regions erroneously labeled as background. As the number of misclassified voxels was small in these cases we classified errors in these slices with a score of 2, to avoid introducing a bias because of the choice of performing this evaluation on coronal slices.

### 2.8. Statistical Analysis of the Hippocampal Volumes

Using the trained CNNs, we labeled every MRI volume in our datasets (220 for EpiBioS4Rx and 424 for EPITARGET). As a demonstration of the applicability of the segmentation, we studied the effects of TBI on hippocampal volume through time and across both hippocampi using a repeated measures linear model, implemented using the linear mixed model function in IBM SPSS Statistics for Windows, version 26.0 (SPSS Inc., Chicago, IL, United States). Every variable was considered as a fixed effect and we assumed a diagonal covariance structure of the error term. Let *t* indicate the time point in days as a scalar variable, *R* be defined such so that *R* = 1 indicates the ipsilateral hippocampus and *R* = 0 the contralateral hippocampus. Additionally, let *B* = 1 indicate the presence of TBI, with *B* = 0 indicating sham animals, and let *E* be the error term. Then, our linear model for the volume *V* can be written as:


(11) $$\begin{array}{l} V=\alpha +\beta_{t}t+\beta_{R}R+\beta_{B}B+\beta_{tR}tR+\beta_{tB}tB+\beta_{RB}RB+E, \end{array}$$

where α, β_*i*_ are parameters of the model.

## 3. Results

**Figure 2:**
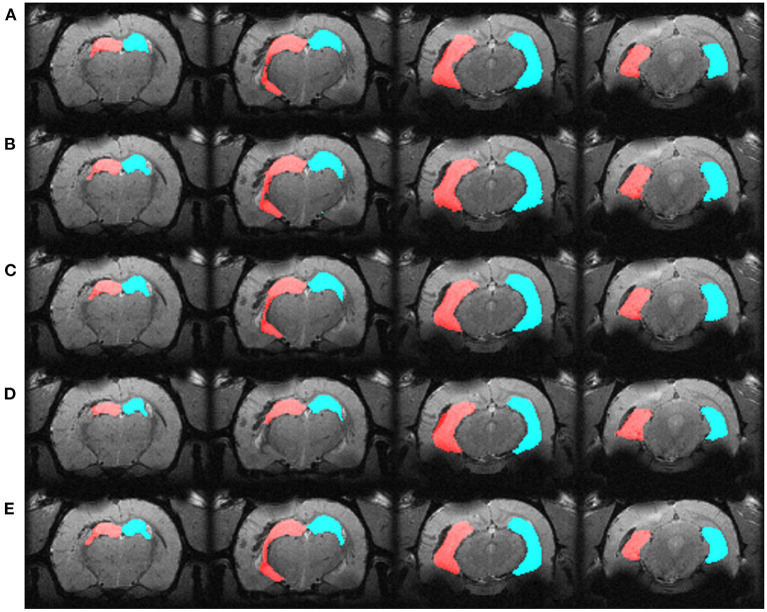
Segmentation maps in four representative slices from a randomly selected animal from the EpiBioS4Rx dataset, at the 2-days timepoint. Segmentation maps were obtained with: **(A)** MU-Net; **(B)** STEPS, **(C)** Majority voting, **(D)** Single-atlas segmentation. **(E)** Displays the ground-truth segmentation. From left to right, slices are located at approximately −2.2, −3.3, −5.0, −6.2 *mm* from bregma. Red: hippocampus ipsilateral to the lesion; blue: hippocampus contralateral to the lesion.

**Figure 3:**
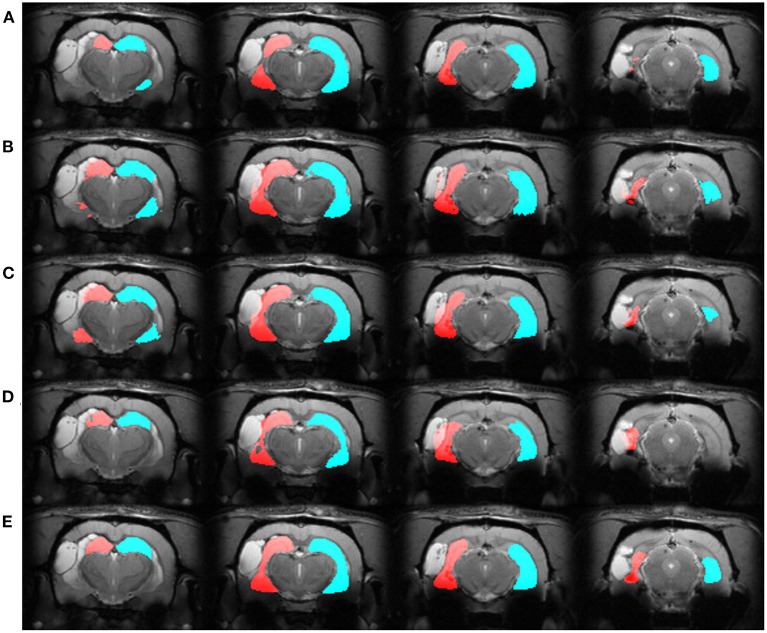
Segmentation maps in four representative slices from a randomly selected animal from the EPITARGET dataset, at the 21-days timepoint. Maps are obtained with: **(A)** MU-Net; **(B)** STEPS, **(C)** Majority voting. **(D)** Single-atlas segmentation. **(E)** Displays the ground-truth segmentation. From left to right, slices are located at approximately −3.5, −4.5, −5.5, −6.5 *mm* from bregma. Red: hippocampus ipsilateral to the lesion; blue: hippocampus contralateral to the lesion. Note how in this case all methods except MU-Net-R mislabel a portion of the lesion as hippocampus.

We automatically annotated every manually-labeled image in the EpiBioS4Rx and EPITARGET datasets using multi-atlas segmentation (STEPS and majority voting), single atlas segmentation, and MU-Net-R. On a qualitative level, both multi-atlas methods and MU-Net-R showed visually convincing segmentation maps, while single-atlas segmentation resulted in the lowest-quality results (Figures 2, 3). Where the hippocampus was markedly displaced by the injury, we noticed that registration-based methods could mislabel the lesioned area as hippocampus, as displayed in Figure 3.

### 3.1. EpiBioS4Rx Segmentation

**Figure 4:**
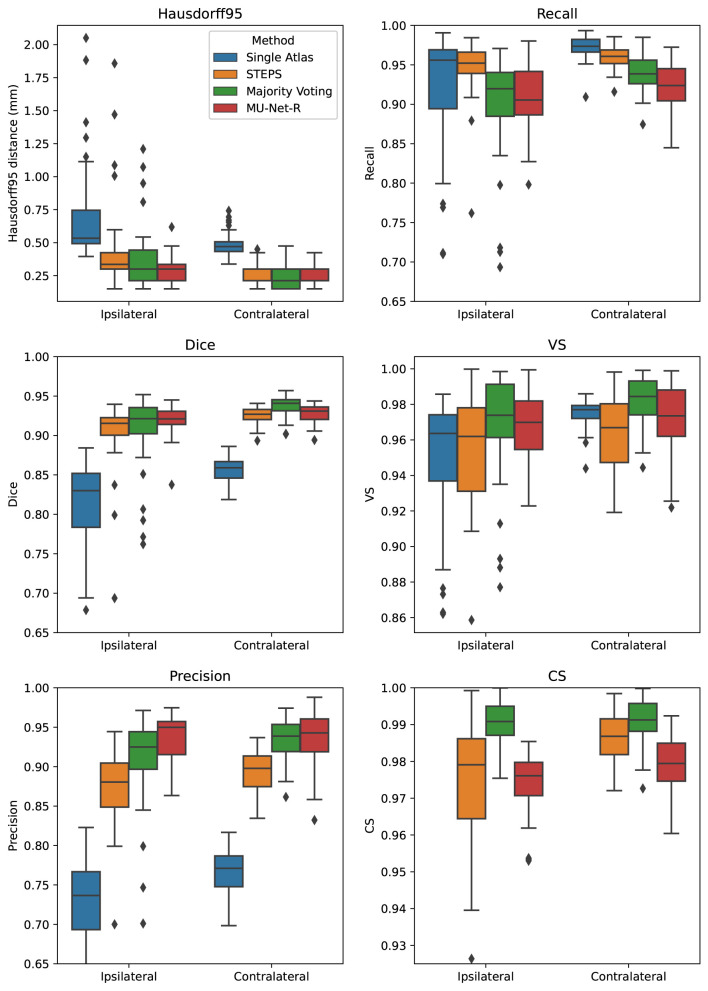
Box plots of all measured quality metrics for the contralateral and ipsilateral hippocampus in the EpiBioS4Rx dataset. Single atlas CS measures (not displayed) average at 0.60 for both hippocampi with a standard deviation of 0.01. Contrary to single-atlas segmentation, MU-Net-R and multi-atlas methods achieve human-level performance.

MU-Net obtained excellent segmentation evaluation scores in both hemispheres as illustrated in Figure 4. For the ipsilateral and contralateral hippocampus MU-Net achieved, respectively, average Dice scores of 0.921 and 0.928, HD95 distances of [0.30] and 0.26 *mm*, precision of 0.935 and 0.936, recall of 0.909 and 0.921, VS of 0.968 and 0.971, and CS of 0.974 and 0.979.

Quantitatively, we observed a marked difference in performance between single-atlas segmentation and all other methods in terms of Dice score, Precision, HD95, and CS (*p* < 0.001 for all tests), with single-atlas segmentation obtaining the worst scores. The performance measures of all other methods were excellent. In terms of HD95 distance, the best performing method was MU-Net on the ipsilateral hemisphere, and majority voting in the contralateral one (*p* < 0.05 for both tests). The same pattern held for the Dice score. MU-Net achieved the highest precision in the ipsilateral hippocampus (*p* < 0.05). We found no significant difference between the precision of MU-Net and that of majority voting in the contralateral one (*p* > 0.7). The precision of both methods was markedly higher than STEPS and single atlas (*p* < 0.05). As an exception to the general trend, single-atlas segmentation showed the highest value of the recall metric in the contralateral hippocampus (*p* < 0.001), while STEPS outperformed it in the ipsilateral one (*p* < 0.02). We found again no significant difference (*p* > 0.8) in the VS scores for majority voting and MU-Net in the ipsilateral hippocampus, with these two methods achieving the highest VS scores (*p* < 0.05). In contrast, majority voting achieved higher VS in the contralateral hemisphere (*p* < 0.0005). Majority voting also better preserved the compactness properties of the hippocampal shape, achieving the highest CS among all the methods (*p* < 0.0005).

### 3.2. EPITARGET Segmentation

**Figure 5:**
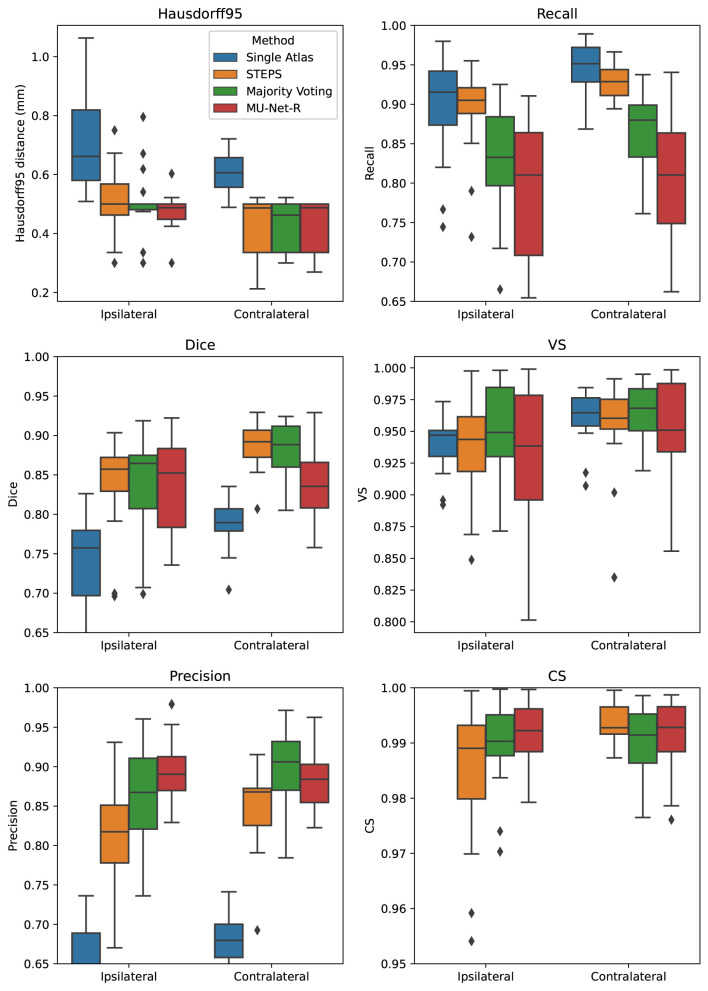
Box plots of all measured quality metrics for the contralateral and ipsilateral hippocampus in the EPITARGET dataset. Single atlas CS measures (not displayed) average at 0.67 for both hippocampi with a standard deviation of 0.02. While MU-Net-R and multi-atlas methods still outperform single-atlas segmentation, in this anisotropic dataset, training MU-Net-R with a smaller dataset, we register a lower performance compared to the EpiBioS4Rx results.

For the EPITARGET dataset, we observed similar pattern of the segmentation evaluation metrics to the ones of EpiBioS4Rx, bilaterally recording good performance metrics for MU-Net-R (see Figure 5). We measured, respectively, for the ipsilateral and contralateral hippocampus, Dice scores of 0.836 and 0.838, HD95 distances of [0.46] and 0.43 *mm*, precision of 0.897 and 0.881, recall of 0.787 and 0.804, VS of 0.928 and 0.946, and CS of 0.992 and 0.991.

In terms of HD95 distance, on the contralateral hemisphere, we found a significant difference between the under-performing single-atlas method and all other methods (*p* < 0.0005), all the other methods performing similarly according to HD95 metric. For the ipsilateral hemisphere, we additionally observed a small advantage for MU-Net-R over STEPS (*p* < 0.05). Similarly, there was no significant Dice score difference in the ipsilateral hippocampus between MU-Net-R and multi-atlas methods. Interestingly, for the contralateral hippocampus, both STEPS and majority voting achieved higher Dice scores than in the lesioned hemisphere (*p* < 0.001), obtaining also a higher Dice score than MU-Net-R (*p* < 0.03). In contrast, MU-Net-R produced similar Dice scores in the two hemispheres (*p* > 0.7). We measured higher precision for majority voting and MU-Net-R compared to all other methods (*p* < 0.001) and no significant difference between the two in both the contralateral and ipsilateral hippocampus (*p* > 0.1). We observed higher recall bilaterally for single atlas segmentation compared to all other methods (*p* < 0.01), with the exception of STEPS on the ipsilateral hemisphere, where the difference was not significant (*p* > 0.2). No significant difference was also detected for VS between the different methods (*p* > 0.1) and for CS on the contralateral hippocampus. Conversely, MU-Net-R performed better than STEPS on the ipsilateral hippocampus (*p* < 0.05).

### 3.3. Inter-hemispheric Differences

**Figure 6:**
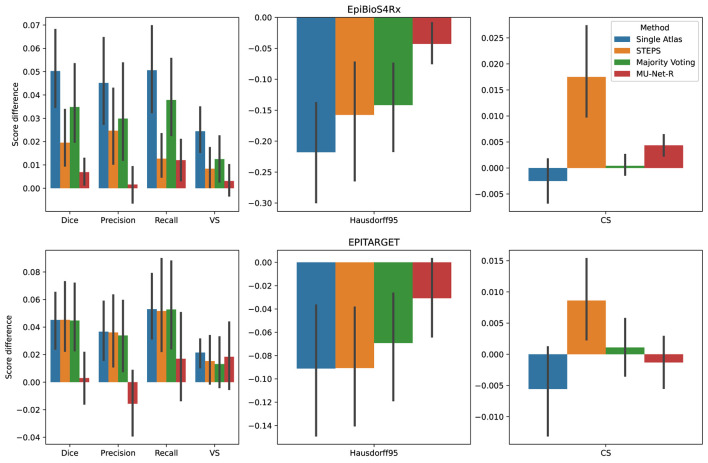
Mean inter-hemispheric differences for all evaluation metrics in single-atlas, STEPS, Majority Voting and MU-Net-R segmentation. Error bars correspond to 95% bootstrapped confidence intervals for the mean. MU-Net-R minimizes inter-hemispheric performance differences across all metrics and on both the EpiBioS4Rx and EPITARGET datasets, with the exception of CS on the EpiBioS4Rx dataset.

We compared the quality of the automatic segmentations, by comparing the evaluation metrics of Section 2.5 between the ipsi and contralateral hemispheres, finding that all segmentation methods obtained better results on the contralateral hippocampus. However, the inter-hemispheric differences for each metric (defined as the average difference in each metric for each brain between the segmentation of the ipsilateral and the contralateral hippocampus) were the smallest for MU-Net-R (Figure 6). MU-Net-R achieved significantly smaller inter-hemispheric differences than other methods in all metrics (maximal *p* < 0.02) with the exception of recall, where both MU-Net-R and STEPS performed better than all other methods (*p* < 0.03), and CS, where majority voting compares favorably to both STEPS and MU-Net-R (maximal *p* < 0.02).

For the EPITARGET dataset and for all evaluation metrics, we observed a smaller average amplitude of the inter-hemispheric differences for MU-Net-R than for other segmentation methods (Figure 6), with a single exception of CS for majority voting. However, differences were statistically significant only for the Dice score (maximal *p* < 0.02), where MU-Net-R demonstrated a stable performance between the two hemispheres. In this case, the average Dice score difference of MU-Net-R was 0.003 with a standard deviation of 0.040, while all other methods displayed an average difference of 0.045 and standard deviations of at least 0.044.

### 3.4. Segmentation Time

The inference time of MU-Net-R was lower than one second per volume. The training of one ensemble of MU-Net-Rs with early stopping required on average 124 min for EpiBioS4Rx and 64 min for EPITARGET. Registering a single volume pair required approximately 40 min for EpiBioS4Rx volumes and 6 min for EPITARGET volumes. After all volumes were registered, applying majority voting and STEPS label fusion required approximately 10 seconds per target volume. Thus, multi-atlas segmentation with 10 atlases required 400 min for EpiBioS4Rx and multi-atlas segmentation with five atlases required 30 min for EPITARGET.

### 3.5. Visual Evaluation

**Figure 7:**
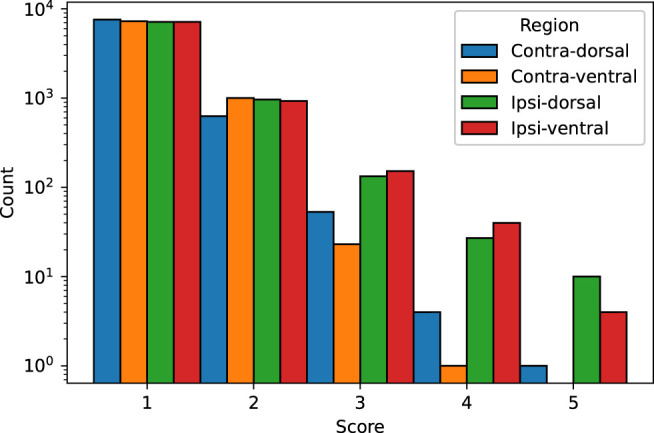
Qualitative score distribution for both hippocampi, divided for the dorsal and ventral aspects of the contralateral and ipsilateral hippocampus. Counts are reported on a logarithmic scale. The overwhelming majority of hippocampal regions were labeled as requiring no corrections (1), followed by regions requiring minor corrections only (2).

**Figure 8:**
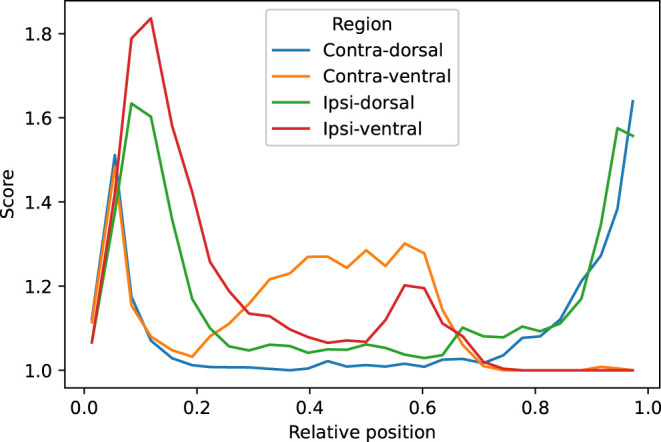
Average qualitative scores as a function of the relative position of the slice across the hippocampus, with 0 indicating the most caudal and 1 the most rostral coronal slice. Averages were obtained by dividing the interval in 30 bins. The vast majority of inaccuracies are located in the most rostral and caudal slices.

We visually evaluated 33136 slices from the 220 volumes in the EpiBioS4Rx dataset as described in Section 2.7. The quality of the MU-Net-R segmentation in the ventral and dorsal aspects of both hippocampi was evaluated on a scale of 1–5, with 1 representing an accurate segmentation and 5 indicating a complete lack of resemblance to the anatomical structure, as outlined in Section 2.7. In the vast majority of cases, the reported score was 1 (Acceptable “as is”), with a small reduction in accuracy for the ipsilateral hippocampus (Figure 7). Overall, we found that 88.00% hippocampal regions were labeled as 1, 10.64% labeled as 2, 1.09% labeled as 3, 0.22% labeled as 4, and 0.05% labeled as 5. As illustrated in Figure 8, the accuracy of the segmentation was the lowest in the most rostral and most caudal coronal slices. Supplementary Figure 6 provides examples of segmented slices of each score.

### 3.6. Hippocampal Volumes

**Figure 9:**
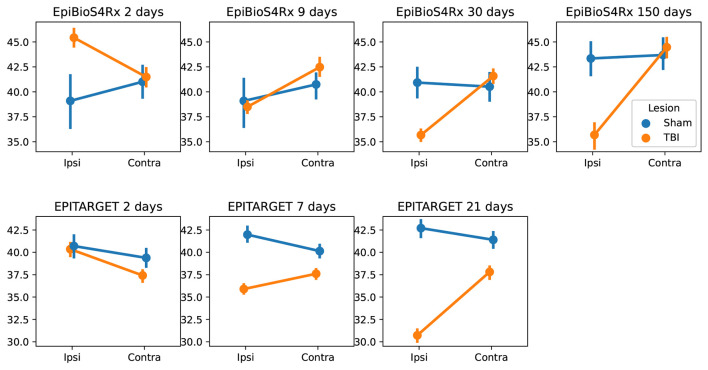
Comparison between the hippocampal volumes (mm^3^) for each dataset and time point between sham and TBI animals. Note the different dynamics in ipsilateral hippocampal volume changes between the EpiBioS4Rx and EPITARGET animals, particularly at 2 days post-injury. The rats in the EpiBioS4Rx cohort were anesthetized with 4% isoflurane at the time of injury. The rats in the EPITARGET cohort were anesthetized with pentobarbital-based anesthesia cocktail, which apparently reduced the acute post-impact seizure-related swelling better than isoflurane.

Using MU-Net-R, we annotated every scan in both the EpiBioS4Rx and EPITARGET dataset and modeled hippocampus volumes as outlined in Section 2.8. As displayed in Figure 9, when comparing sham and TBI rats in EpiBioS4Rx we found that all included factors were statistically significant in explaining the volume: lesion status (sham or TBI), timepoint, and ROI, as well as their pairwise interaction terms. With the exception of the presence of lesions (*p* = 0.029), all other *p*-values were smaller or equal to 0.001, for both single factors and interaction terms. The same was true for the EPITARGET dataset, where all factors were highly significant (*p* < 0.002).

Supplementary Table 1 provides the complete SPSS output, including β coefficients, *p*-values, *F*, and *t* statistics.

## 4. Discussion

We designed and trained a CNN-based approach (MU-Net-R) to hippocampal segmentation in rat brain MRI after TBI. We quantitatively evaluated MU-Net-R-based segmentation and compared it with single- and multi-atlas registration-based segmentation methods through a variety of metrics (Dice score, VS, HD, CS, precision, and recall). These evaluations demonstrated that MU-Net-R achieved state-of-the-art performance in terms of segmentation quality while reducing the bias for healthy anatomy, typical of registration-based methods, and with a marked reduction in the segmentation time compared to registration-based methods.

Single-atlas segmentation displayed the least satisfactory results, with a marked decrease in segmentation quality in terms of precision, Dice score, and HD95 distance compared to all other methods. Conversely, STEPS, majority voting, and MU-Net-R displayed excellent performance, with Dice coefficients compatible with human inter-rater agreement (3, 56, 57). Differences in each performance metric between these methods were overall small, and different metrics would indicate a different preference. Thus, a global preference between MU-Net-R, STEPS, and majority voting did not emerge simply from these measures.

In contrast, as illustrated in Figure 6, MU-Net-R segmentation resulted in a marked reduction in the differences between the two hemispheres in each metric quantifying the agreement between the segmentation mask and the respective ground truth. A small bias was still measured, both quantitatively and qualitatively. In our qualitative evaluation of MU-Net-R segmentations, we detected a larger number of slices evaluated with scores of 3 (Moderate edits required, 20–50% of the area would need to be changed) or higher for the ipsilateral hippocampus. This difference was likely owed to the small size of the training set; the presence of lesions manifesting in different shapes and sizes implies a larger degree of variability that requires more training data to capture this variability. Taking this small difference into account, the vast majority of slices required no correction regardless of ROI, with only 2% of slices requiring moderate or larger edits.

A reduced performance of registration-based segmentation methods in the presence of lesions has been documented in human MRI (9). Here, as an additional contribution of our work, we quantified and documented this difference in rat brain MRI with TBI according to six different metrics. This reduced performance might imply the presence of random or systematic errors in terms of the shape or positioning of the segmentation of the ipsilateral hippocampus.

The inference time for MU-Net-R was markedly faster than that of registration-based methods, requiring less than one second for a single MRI volume, whereas multi-atlas segmentation required from 30 to 400 min depending on the dataset. As for training, using the early stopping strategy described in Section 2.3.3, with this dataset size, training one ensemble of CNNs required a comparable amount of time to that required by registration, with the added benefit of obtaining a network that can then be used to quickly label a large number of new volumes. For this reason, our method would be preferable when processing large datasets, or where time constraints could be relevant, for example, in an online system designed to quickly provide a segmentation map to the user. The training time was especially low in the case of the EPITARGET data, where the number of training volumes is smaller, and the limited resolution further reduces the amount of information present in each data sample.

Compared to our previous work (29), MU-Net, the architecture of MU-Net-R was adjusted to reduce the number of parameters to better manage with the small training sets. The size of all kernels was reduced from the 5 × 5 to 3 × 3, and the overall number of convolution operations has been decreased in the first two blocks of the neural network, whereas MU-Net used 64 kernels for each convolution. We further replaced the Dice loss of MU-Net with the generalized Dice loss [see (42)] in the loss function of MU-Net-R. Generalized Dice loss balances the different classes against the background by introducing weights based on the size of ROIs. In analogy with MU-Net, M-Net-R was characterized by comparatively higher precision and lower recall. While this difference was not large [for example as compared to the decrease in precision, Equation (10) for single-atlas segmentation] it may still indicate a bias for the background class, which may not be entirely corrected by the weighting parameters introduced by the generalized Dice loss. The large decrease in precision for single-atlas segmentation was largely corrected by combining multiple atlases through majority voting, although this precision remained lower than that measured for MU-Net-R and came at the price of a lower recall.

Consistently with (58), we observed a very small difference between Dice scores of STEPS and majority voting multi-atlas segmentation, of 0.012 on the contralateral hippocampus and 0.003 on the ipsilateral one. Thus, STEPS and majority voting appear to be largely equal according to this metric. In contrast, in our previous work (29) we measured a larger (0.055) and statistically significant Dice score difference in favor of STEPS over majority voting for the segmentation of the healthy hippocampus. Two possible reasons why this difference is not observed in our case may be found in the different MRI setup, featuring a different coil and generating visibly different images. Furthermore, it could be a consequence of having performed diffeomorphic registration using a different method, FSL FNIRT (59), while the present work implemented registration tools from ANTs (46).

Concerning the per-sample computation time, replacing registration-based methods with CNNs resulted in a vast reduction from 40 min in EpiBioS4Rx and 10 in EPITARGET, to <1 s. This comes at the price of an initial training procedure requiring, respectively, 124 and 64 min. In those applications where the labeled templates and the target images are sampled from the same distribution then MU-Net-R becomes more time-efficient compared to applying registration-based methods for datasets larger than six samples.

Thanks to the segmentation maps generated by the CNN, we were able to segment the entire EpiBioS4Rx and EPITARGET datasets in a reasonable time. To exemplify a data analysis pipeline, we evaluated the effect of the presence of the lesion on the volume of both hippocampi, both in general and combined with timepoint and ROI. The presence of TBI was an important predictor of hippocampal volume both as a single factor and in combination with timepoint and ROI, indicating that the volume of both the ipsilateral and contralateral hippocampus changed as a consequence of TBI.

Even though the size of the entire datasets were larger than in most previous preclinical studies of TBI, the training datasets were small due to the cost of manual segmentation of MRIs and the modest size of the training sets are one of the limitations of our work. It is reasonable to believe that larger training datasets would further enhance the performance of our neural network. Another unresolved problem is the high degree of specialization of the CNNs to each MRI setup. While this problem can be attacked with a variety of methods, e.g., transfer learning (60), it is still an open question for CNNs.

In this work, we have demonstrated how replacing registration-based methods with CNNs can simultaneously increase the speed of segmentation and obtain more reliable delineations of ROIs in the presence of anatomical alterations due to brain injury. Although we have limited our analysis to a specific network architecture, we do not assume that this robustness to anatomical alternations due to brain injury to be a unique feature of our neural network or even limited to the U-Net-like architectures commonly applied in medical imaging. Because CNNs eliminate reliance on registration and replace it with encoding knowledge in the parameters of the network itself, and given their intrinsic properties of spatial invariance, we expect segmentation CNNs to be generally more robust to anatomical change than registration-based methods.

## Data Availability Statement

The code used in our work is freely available under the MIT license at: GitHub, https://github.com/Hierakonpolis/MU-Net-R. The MRI data is stored on the University of Eastern Finland's servers and will be made available upon request. Requests to access these datasets should be directed to Asla Pitkänen, asla.pitkanen@uef.fi; Jussi Tohka, jussi.tohka@uef.fi.

## Ethics Statement

All experiments were approved by the Animal Ethics Committee of the Provincial Government of Southern Finland and were performed in accordance with the guidelines of the European Community Directives 2010/63/EU.

## Author Contributions

RD: methodology, software, formal analysis, investigation, and writing—original draft. EH: data curation and methodology. EM: software and investigation. RI: investigation. JV: methodology, writing—review, and editing. XN-E: data curation, formal analysis, and writing—original draft. OG: conceptualization. AP and JT: conceptualization, methodology, and writing—original draft. All authors contributed to the article and approved the submitted version.

## Funding

This study was supported by the Medical Research Council and Natural Science and Engineering Research Council of the Academy of Finland (Grants 272249, 273909, and 2285733-9 to AP, 316258 to JT) and by the European Union's Seventh Framework Programme (FP7/2007-2013) under grant agreement n°602102 (EPITARGET) (AP), the National Institute of Neurological Disorders and Stroke (NINDS) Centres without Walls (grant number U54 NS100064) (AP), the Sigrid Juselius Foundation (AP), by grant S21770 from the European Social Fund (JT), grant 65211916 from the North Savo Regional Fund (RD), from the European Union's Horizon 2020 Framework Programme [Marie Sklodowska Curie grant agreement 740264 (GENOMMED)] (JV), and by the Alfred Kordelin Foundation (EM).

## Conflict of Interest

The authors declare that the research was conducted in the absence of any commercial or financial relationships that could be construed as a potential conflict of interest.

## Publisher's Note

All claims expressed in this article are solely those of the authors and do not necessarily represent those of their affiliated organizations, or those of the publisher, the editors and the reviewers. Any product that may be evaluated in this article, or claim that may be made by its manufacturer, is not guaranteed or endorsed by the publisher.

## Abbreviations

BET: brain extraction tool
CNN: convolutional neural network
CS: compactness score
HD95: 95th percentile of the Hausdorff distance
MR: magnetic resonance
MRI: magnetic resonance imaging
ROI: region of interest
SyN: symmetric image normalization
STEPS: similarity and truth estimation for propagated segmentation
TBI: traumatic brain injury
VS: volume similarity.

## References

[1] Anderson RJ Cook JJ Delpratt N Nouls JC Gu B McNamara JO. Small animal multivariate brain analysis (SAMBA)–a high throughput pipeline with a validation framework. Neuroinformatics. (2019) 17:451–72. 10.1007/s12021-018-9410-0 30565026 PMC6584586

[2] Calabrese E Badea A Cofer G Qi Y Johnson GA. A diffusion MRI tractography connectome of the mouse brain and comparison with neuronal tracer data. Cereb Cortex. (2015) 25:4628–37. 10.1093/cercor/bhv121 26048951 PMC4715247

[3] Ali AA Dale AM Badea A Johnson GA. Automated segmentation of neuroanatomical structures in multispectral MR microscopy of the mouse brain. Neuroimage. (2005) 27:425–35. 10.1016/j.neuroimage.2005.04.017 15908233

[4] De Feo R Giove F. Towards an efficient segmentation of small rodents brain: a short critical review. J Neurosci Methods. (2019) 323:82–9. 10.1016/j.jneumeth.2019.05.003 31102669

[5] Pagani M Damiano M Galbusera A Tsaftaris SA Gozzi A. Semi-automated registration-based anatomical labelling, voxel based morphometry and cortical thickness mapping of the mouse brain. J Neurosci Methods. (2016) 267:62–73. 10.1016/j.jneumeth.2016.04.007 27079699

[6] Iglesias JE Sabuncu MR. Multi-atlas segmentation of biomedical images: a survey. Med Image Anal. (2015) 24:205–19. 10.1016/j.media.2015.06.012 26201875 PMC4532640

[7] Cardoso MJ Leung K Modat M Keihaninejad S Cash D Barnes J. STEPS: Similarity and truth estimation for propagated Segmentations and its application to hippocampal segmentation and brain parcelation. Med Image Anal. (2013) 17:671–84. 10.1016/j.media.2013.02.006 23510558

[8] Immonen RJ Kharatishvili I Niskanen JP Gröhn H Pitkänen A Gröhn OH. Distinct MRI pattern in lesional and perilesional area after traumatic brain injury in rat–11 months follow-up. Exp Neurol. (2009) 215:29–40. 10.1016/j.expneurol.2008.09.009 18929562

[9] Despotović I Goossens B Philips W. MRI segmentation of the human brain: challenges, methods, and applications. Comput Mathemat Methods Med. (2015). 2015:1–23. 10.1155/2015/450341 25945121 PMC4402572

[10] Ledig C Heckemann RA Hammers A Lopez JC Newcombe VF Makropoulos A. Robust whole-brain segmentation: application to traumatic brain injury. Med Image Anal. (2015) 21:40–58. 10.1016/j.media.2014.12.003 25596765

[11] Roy S Butman JA Pham DL Alzheimers Disease Neuroimaging Initiative. Robust skull stripping using multiple MR image contrasts insensitive to pathology. Neuroimage. (2017) 146:132–47. 10.1016/j.neuroimage.2016.11.017 27864083 PMC5321800

[12] Shao M Han S Carass A Li X Blitz AM Shin J. Brain ventricle parcellation using a deep neural network: application to patients with ventriculomegaly. Neuroimage. (2019) 23:101871. 10.1016/j.nicl.2019.101871 31174103 PMC6551563

[13] Diamond BR Mac Donald CL Frau-Pascual A Snider SB Fischl B Dams-O'Connor K. Optimizing the accuracy of cortical volumetric analysis in traumatic brain injury. MethodsX. (2020) 7:100994. 10.1016/j.mex.2020.100994 32760659 PMC7393399

[14] Pluta J Avants BB Glynn S Awate S Gee JC Detre JA. Appearance and incomplete label matching for diffeomorphic template based hippocampus segmentation. Hippocampus. (2009) 19:565–71. 10.1002/hipo.20619 19437413

[15] Mansoor NM Vanniyasingam T Malone I Hobbs NZ Rees E Durr A. Validating automated segmentation tools in the assessment of caudate atrophy in Huntington's disease. Front Neurol. (2021). 12:616272. 10.3389/fneur.2021.616272 33935934 PMC8079754

[16] O'Shea K Nash R. An introduction to convolutional neural networks. arXiv Preprint. (2015) arXiv:151108458.

[17] Ronneberger O Fischer P Brox T. U-net: convolutional networks for biomedical image segmentation. In: International Conference on Medical Image Computing and Computer-Assisted Intervention. Singapore (2015). p. 234–41.

[18] Milletari F Navab N Ahmadi SA. V-net: fully convolutional neural networks for volumetric medical image segmentation. In: 2016 Fourth International Conference on 3D Vision (3DV). IEEE. Stanford, CA, (2016). p. 565–71.

[19] Badrinarayanan V Kendall A Cipolla R. Segnet: a deep convolutional encoder-decoder architecture for image segmentation. IIEEE Trans Pattern Anal Mach Intell. (2017) 39:2481–95. 10.1109/TPAMI.2016.2644615 28060704

[20] Wachinger C Reuter M Klein T. DeepNAT: Deep convolutional neural network for segmenting neuroanatomy. Neuroimage. (2018) 170:434–45. 10.1016/j.neuroimage.2017.02.035 28223187 PMC5563492

[21] Oktay O Schlemper J Folgoc LL Lee M Heinrich M Misawa K. Attention u-net: learning where to look for the pancreas. arXiv Preprint. (2018) arXiv:180403999.

[22] Rundo L Han C Nagano Y Zhang J Hataya R Militello C. USE-Net: incorporating Squeeze-and-Excitation blocks into U-Net for prostate zonal segmentation of multi-institutional MRI datasets. Neurocomputing. (2019) 365:31–43. 10.1016/j.neucom.2019.07.006

[23] Isola P Zhu JY Zhou T Efros AA. Image-to-image translation with conditional adversarial networks. In: Proceedings of the IEEE Conference on Computer Vision and Pattern Recognition. San Juan, PR (2017). p. 1125–34.

[24] Pelt DM Sethian JA. A mixed-scale dense convolutional neural network for image analysis. Proc Natl Acad Sci USA. (2018) 115:254–9. 10.1073/pnas.1715832114 29279403 PMC5777062

[25] Roy S Knutsen A Korotcov A Bosomtwi A Dardzinski B Butman JA. A deep learning framework for brain extraction in humans and animals with traumatic brain injury. In: 2018 IEEE 15th International Symposium on Biomedical Imaging (ISBI 2018). Washington, DC (2018). p. 687–91.

[26] Hsu LM Wang S Ranadive P Ban W Chao THH Song S. Automatic skull stripping of rat and mouse brain MRI data using U-net. Front Neurosci. (2020) 14:568614. 10.3389/fnins.2020.568614 33117118 PMC7575753

[27] Valverde JM Shatillo A De Feo R Gröhn O Sierra A Tohka J. RatLesNetv2: a fully convolutional network for rodent brain lesion segmentation. Front Neurosci. (2020) 14:1333. 10.3389/fnins.2020.610239 33414703 PMC7783408

[28] Valverde JM Shatillo A De Feo R Gröhn O Sierra A Tohka J. Automatic rodent brain MRI lesion segmentation with fully convolutional networks. In: International Workshop on Machine Learning in Medical Imaging. Strasbourg (2019). p. 195–202.

[29] De Feo R Shatillo A Sierra A Valverde JM Gröhn O Giove F. Automated joint skull-stripping and segmentation with Multi-Task U-Net in large mouse brain MRI databases. NeuroImage. (2021) 2021:117734. 10.1016/j.neuroimage.2021.117734 33454412

[30] Immonen R Smith G Brady RD Wright D Johnston L Harris NG. Harmonization of pipeline for preclinical multicenter MRI biomarker discovery in a rat model of post-traumatic epileptogenesis. Epilepsy Res. (2019) 150:46–57. 10.1016/j.eplepsyres.2019.01.001 30641351 PMC6818721

[31] Ndode-Ekane XE Santana-Gomez C Casillas-Espinosa PM Ali I Brady RD Smith G. Harmonization of lateral fluid-percussion injury model production and post-injury monitoring in a preclinical multicenter biomarker discovery study on post-traumatic epileptogenesis. Epilepsy Res. (2019) 151:7–16. 10.1016/j.eplepsyres.2019.01.006 30711714 PMC6812686

[32] Lapinlampi N Andrade P Paananen T Hämäläinen E Ekolle Ndode-Ekane X Puhakka N. Postinjury weight rather than cognitive or behavioral impairment predicts development of posttraumatic epilepsy after lateral fluid-percussion injury in rats. Epilepsia. (2020) 61:2035–52. 10.1111/epi.16632 32786029

[33] Manninen E Chary K Lapinlampi N Andrade P Paananen T Sierra A. Early increase in cortical T2 relaxation is a prognostic biomarker for the evolution of severe cortical damage, but not for epileptogenesis, after experimental traumatic brain injury. J Neurotrauma. (2020) 37:2580–94. 10.1089/neu.2019.6796 32349620

[34] Immonen R Kharatishvili I Gröhn O Pitkänen A. MRI biomarkers for post-traumatic epileptogenesis. J Neurotrauma. (2013) 30:1305–9. 10.1089/neu.2012.2815 23469770 PMC3713441

[35] Kharatishvili I Immonen R Gröhn O Pitkänen A. Quantitative diffusion MRI of hippocampus as a surrogate marker for post-traumatic epileptogenesis. Brain. (2007) 130:3155–68. 10.1093/brain/awm268 18055492

[36] Paxinos G Watson C. The Rat Brain in Stereotaxic Coordinates. 6th ed. Amsterdam; Boston, MA: Academic Press/Elsevier (2007).

[37] Van der Walt S Schönberger JL Nunez-Iglesias J Boulogne F Warner JD Yager N. scikit-image: image processing in Python. PeerJ. (2014) 2:e453. 10.7717/peerj.453 25024921 PMC4081273

[38] Noh H Hong S Han B. Learning deconvolution network for semantic segmentation. In: Proceedings of the IEEE International Conference on Computer Vision (2015). p. 1520–8. 10.1109/ICCV.2015.17827295638

[39] Maas AL Hannun AY Ng AY. Rectifier nonlinearities improve neural network acoustic models. In: Proceedings of the 30 th International Conference on Machine Learning,JMLR: W&CP, Vol. 28 Atlanta, GA (2013).

[40] Ioffe S Szegedy C. Batch normalization: accelerating deep network training by reducing internal covariate shift. arXiv Preprint. (2015) arXiv:150203167.

[41] Isensee F Jaeger PF Kohl SA Petersen J Maier-Hein KH. nnU-Net: a self-configuring method for deep learning-based biomedical image segmentation. Nat Methods. (2021) 18:203–11. 10.1038/s41592-020-01008-z 33288961

[42] Sudre CH Li W Vercauteren T Ourselin S Cardoso MJ. Generalised Dice overlap as a deep learning loss function for highly unbalanced segmentations. In: Deep Learning in Medical Image Analysis and Multimodal Learning for Clinical Decision Support. Granada (2017). p. 240–8. 10.1007/978-3-319-67558-9_28 PMC7610921 34104926

[43] Liu L Jiang H He P Chen W Liu X Gao J. On the variance of the adaptive learning rate and beyond. arXiv Preprint. (2019) arXiv:190803265. 25398893

[44] Kingma DP Ba J. Adam: a method for stochastic optimization. arXiv Preprint. (2014) arXiv:14126980.

[45] Virtanen P Gommers R Oliphant TE Haberland M Reddy T Cournapeau D. SciPy 1.0: fundamental algorithms for scientific computing in Python. Nat Methods. (2020) 17:261–72. 10.1038/s41592-020-0772-5 32015543 PMC7056644

[46] Avants BB Tustison NJ Song G Cook PA Klein A Gee JC. A reproducible evaluation of ANTs similarity metric performance in brain image registration. Neuroimage. (2011) 54:2033–44. 10.1016/j.neuroimage.2010.09.025 20851191 PMC3065962

[47] Smith SM. Fast robust automated brain extraction. Hum Brain Mapp. (2002) 17:143–55. 10.1002/hbm.10062 12391568 PMC6871816

[48] Cardoso MJ Modat M Ourselin S Keihaninejad S Cash D. STEPS: multi-label similarity and truth estimation for propagated segmentations. In: 2012 IEEE Workshop on Mathematical Methods in Biomedical Image Analysis (MMBIA). San Francisco, CA (2012). p. 153–8.

[49] Dice LR. Measures of the amount of ecologic association between species. Ecology. (1945) 26:297–302. 10.2307/1932409

[50] Huttenlocher DP Klanderman GA Rucklidge WJ. Comparing images using the Hausdorff distance. IEEE Trans Pattern Anal Mach Intell. (1993) 15:850–63. 10.1109/34.23207327295638

[51] Maier O Rothberg A Raamana PR Bges R Isensee F Ahern M. loli/medpy: MedPy 0.4.0. Zenodo (2019). 10.5281/zenodo.2565940

[52] Taha AA Hanbury A. Metrics for evaluating 3D medical image segmentation: analysis, selection, and tool. BMC Med Imaging. (2015) 15:1–28. 10.1186/s12880-015-0068-x 26263899 PMC4533825

[53] Bribiesca E. An easy measure of compactness for 2D and 3D shapes. Pattern Recognit. (2008) 41:543–54. 10.1016/j.patcog.2007.06.029

[54] Chung E Romano JP. Exact and asymptotically robust permutation tests. Ann Statist. (2013) 41:484–507. 10.1214/13-AOS109018320210

[55] Greenham S Dean J Fu CKK Goman J Mulligan J Tune D. Evaluation of atlas-based auto-segmentation software in prostate cancer patients. J Med Radiat Sci. (2014) 61:151–8. 10.1002/jmrs.64 26229651 PMC4175851

[56] Yushkevich PA Piven J Hazlett HC Smith RG Ho S Gee JC. User-guided 3D active contour segmentation of anatomical structures: significantly improved efficiency and reliability. Neuroimage. (2006) 31:1116–28. 10.1016/j.neuroimage.2006.01.015 16545965

[57] Entis JJ Doerga P Barrett LF Dickerson BC. A reliable protocol for the manual segmentation of the human amygdala and its subregions using ultra-high resolution MRI. Neuroimage. (2012) 60:1226–35. 10.1016/j.neuroimage.2011.12.073 22245260 PMC3665767

[58] Bai J Trinh TLH Chuang KH Qiu A. Atlas-based automatic mouse brain image segmentation revisited: model complexity vs. image registration. Magn Reson Imaging. (2012) 30:789–98. 10.1016/j.mri.2012.02.010 22464452

[59] Andersson JL Jenkinson M Smith S. Non-linear Registration Aka Spatial Normalisation FMRIB Technial Report TR07JA2. FMRIB Analysis Group of the University of Oxford (2007).

[60] Valverde JM Imani V Abdollahzadeh A De Feo R Prakash M Ciszek R. Transfer learning in magnetic resonance brain imaging: a systematic review. J Imaging. (2021) 7:66. 10.3390/jimaging7040066 34460516 PMC8321322

