# Supplementary material for: Convolutional Neural Networks Enable Robust Automatic Segmentation of the Rat Hippocampus in MRI After Traumatic Brain Injury
Source: Front Neurol. 2022 Feb 17;13:820267. doi: 10.3389/fneur.2022.820267 (PMC8891699; doi:10.3389/fneur.2022.820267)
Supplement: Supplementary file 1 [file Data_Sheet_1.PDF]

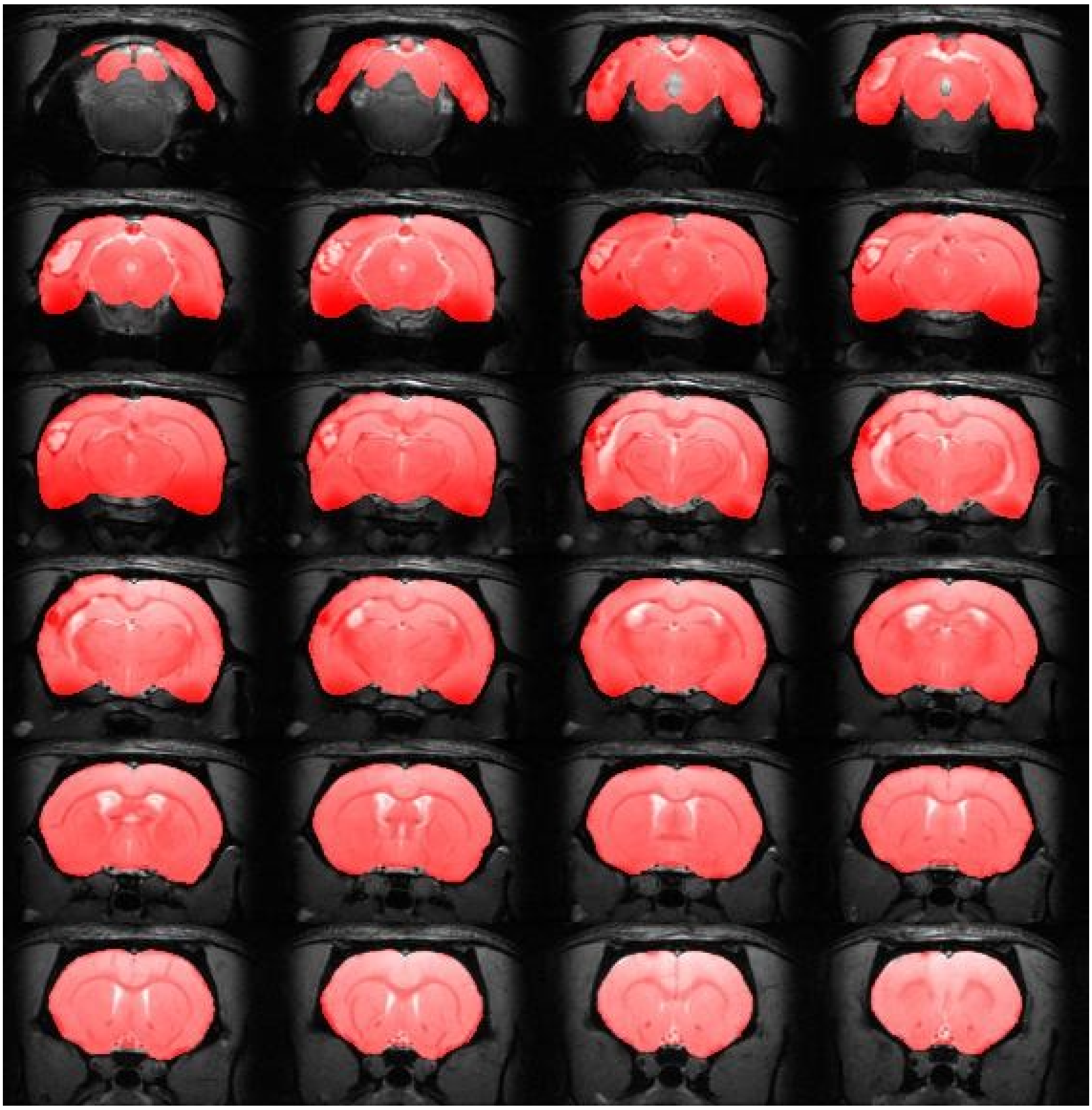

Figure 1: Manual annotation of the brain mask in an EPITARGET rat, MRI scan acquired 21 days after TBI, displaying every annotated coronal slice.

Method	ROI	Dice	HD95 (mm)	VS	CS	Precision	Recall
MU-Net-R	Ipsi	**0.921 ± 0.017**	**0.302 ± 0.092**	0.968 ± 0.019	0.974 ± 0.008	**0.935 ± 0.092**	0.909 ± 0.098
MU-Net	Ipsi	**0.923 ± 0.029**	**0.291 ± 0.109**	0.972 ± 0.016	0.980 ± 0.007	0.926 ± 0.037	0.922 ± 0.045
NE	Ipsi	0.904 ± 0.024	0.335 ± 0.100	0.966 ± 0.020	0.979 ± 0.009	0.921 ± 0.036	0.909 ± 0.049
MU-Net-R	Contra	**0.928 ± 0.011**	**0.259 ± 0.072**	0.971 ± 0.020	0.979 ± 0.007	0.936 ± 0.033	0.921 ± 0.033
MU-Net	Contra	**0.922 ± 0.011**	0.264 ± 0.079	0.973 ± 0.019	0.982 ± 0.007	0.935 ± 0.034	0.931 ± 0.030
NE	Contra	0.914 ± 0.013	0.288 ± 0.077	0.972 ± 0.023	0.981 ± 0.008	0.926 ± 0.0355	0.925 ± 0.036

Table 1: Ablation tests comparing MU-Net-R, MU-Net, and Non-Ensembled MU-Net-R (NE). Bold numbers indicate a significant statistical difference (p<0.05) compared with NE. The architectural choices in MU-Net-R result in statistically equal results compared to MU-Net, with a strong reduction in the number of parameters. Compared to NE, we observe a marked increase in the network's performance as a consequence of ensembling.

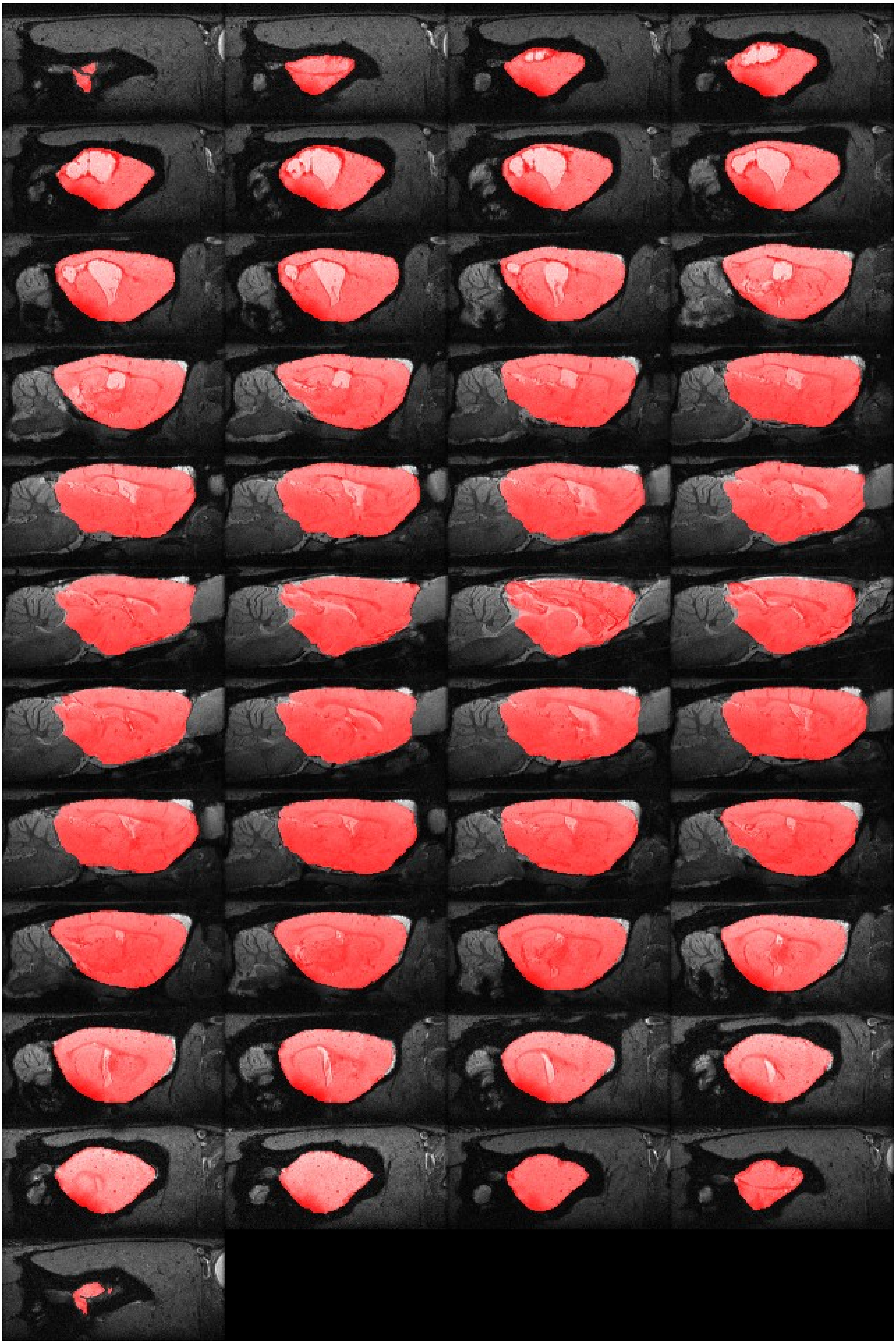

Figure 2: Manual annotation of the brain mask in an EpiBioS4Rx rat, MRI scan acquired 150 days after TBI, displaying every annotated sagittal slice.

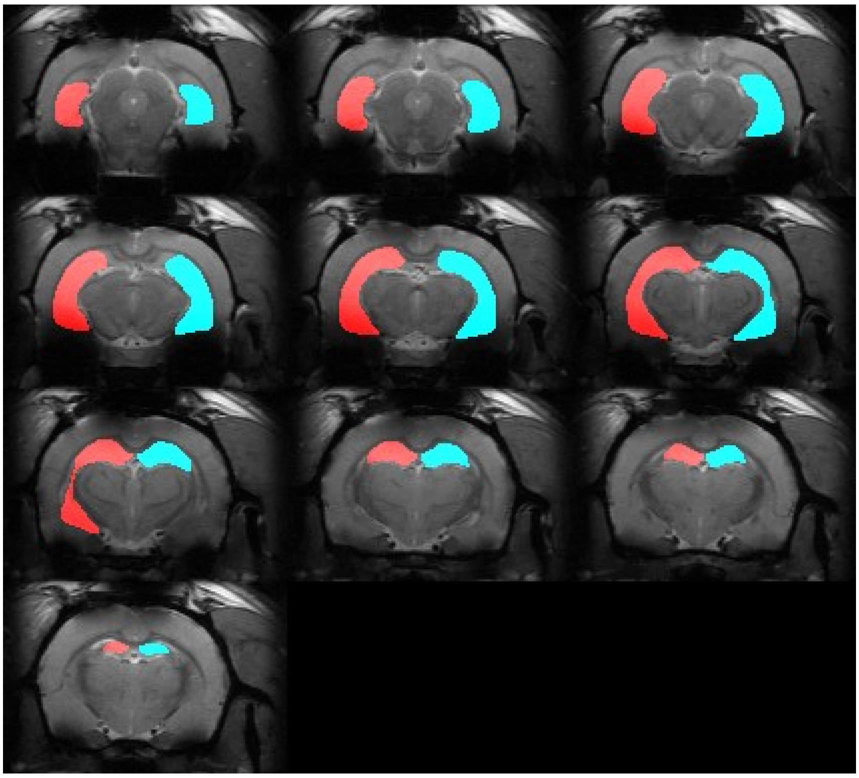

Figure 3: Manual annotation of the hippocampus ipsilateral (red) and contralateral (blue) to the lesion, in an EPITARGET rat two days after TBI.

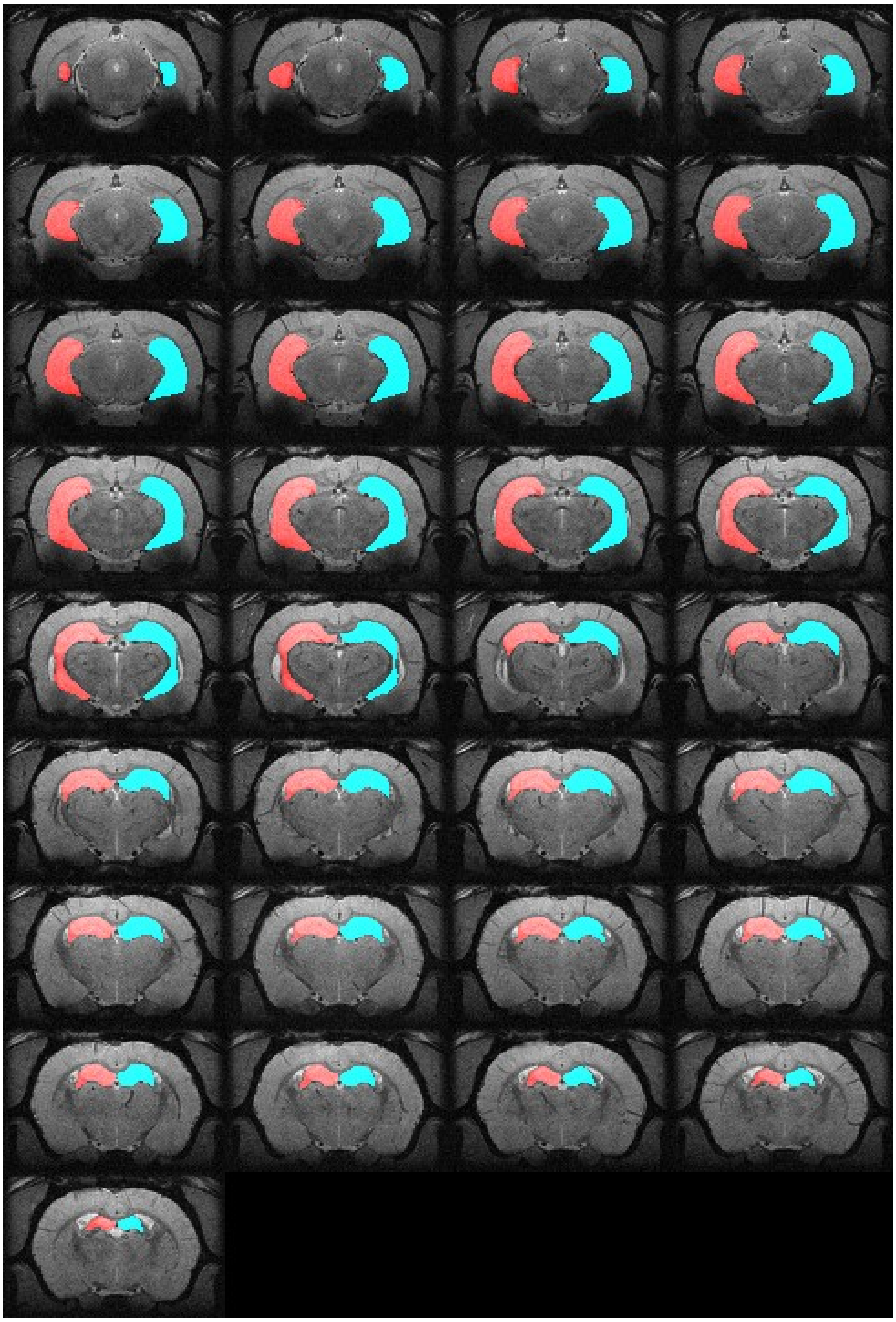

Figure 4: Manual annotation of the hippocampus ipsilateral (red) and contralateral (blue) to the lesion, in an EpiBioS4Rx rat nine days after TBI.

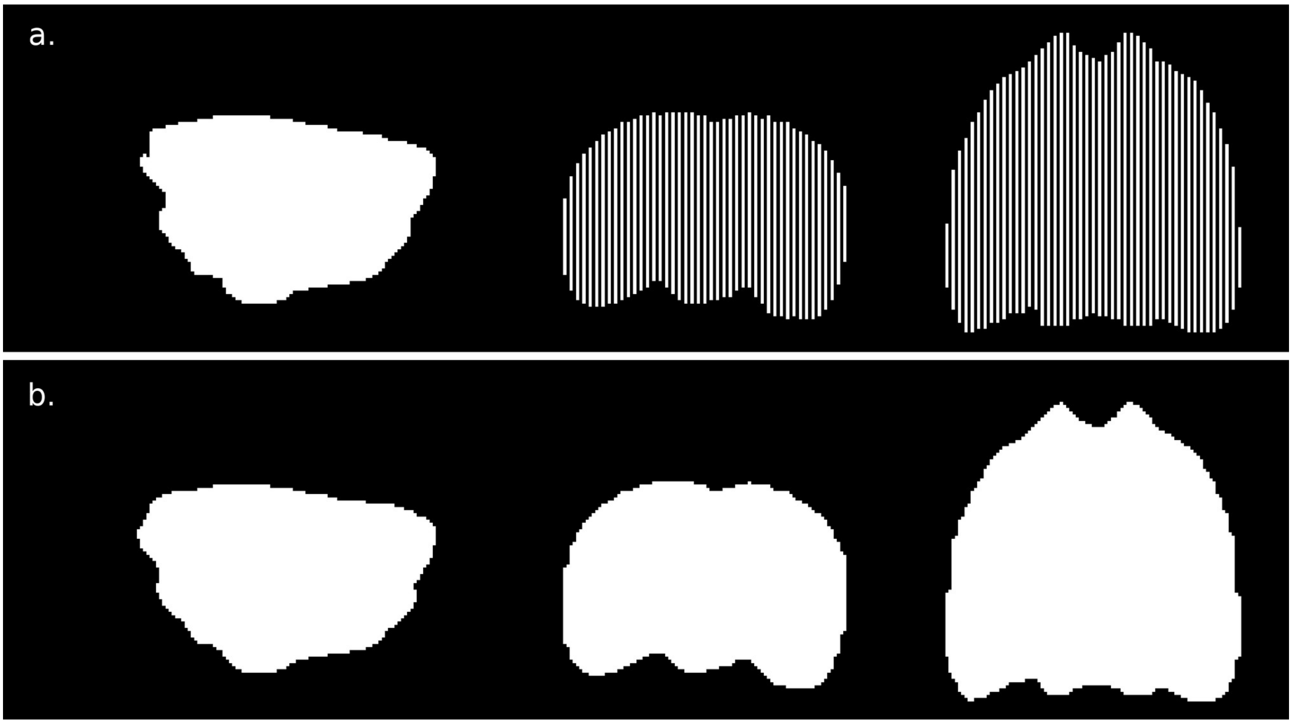

Figure 5: Brain mask completion for the EpiBioS4Rx dataset. a. First, we manually labeled the brain mask in every second sagittal slice. b. Second, we applied a binary-closing operation to obtain a brain mask for the whole brian.

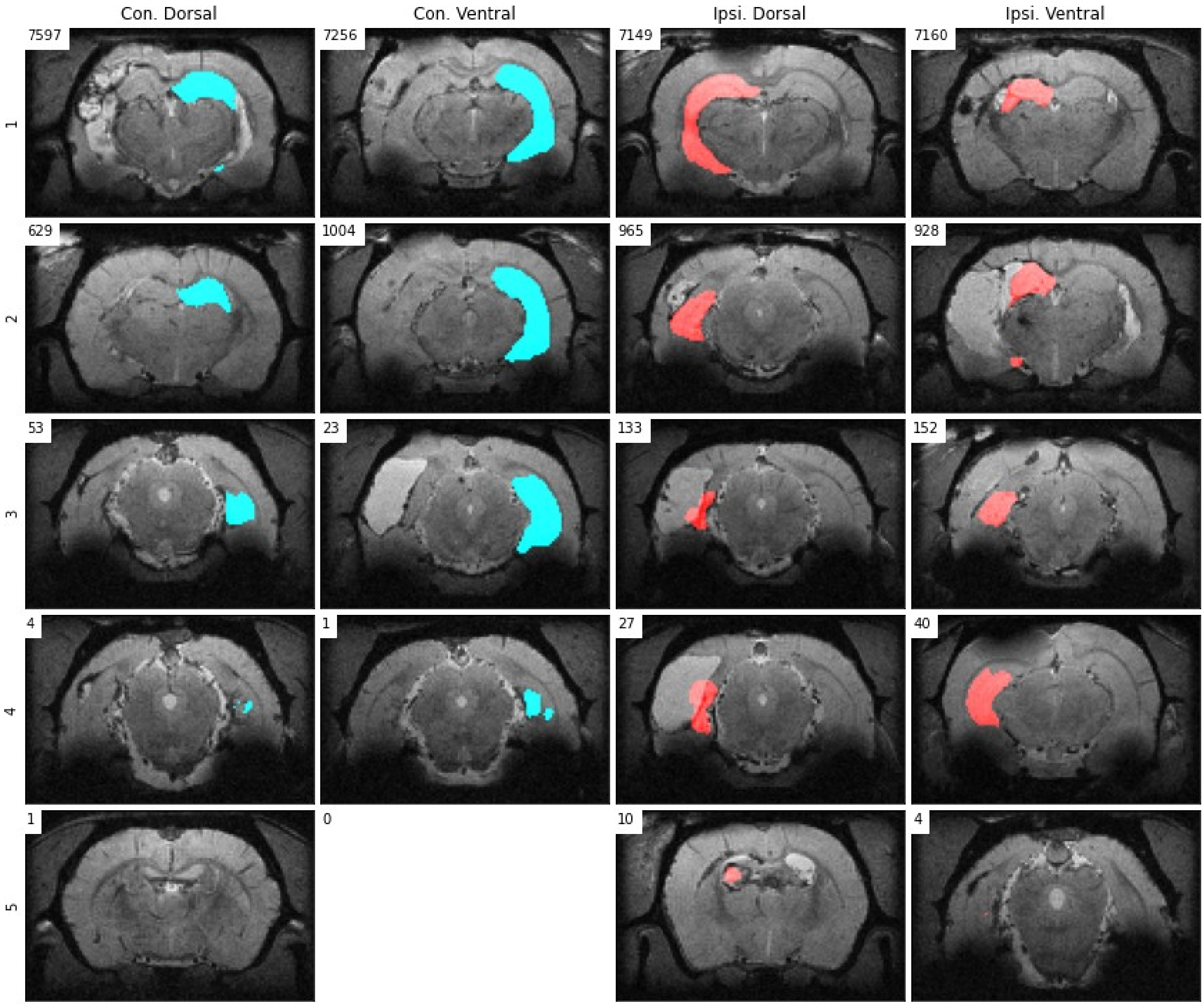

Figure 6: Examples of MU-Net-R hippocampus segmentations and their visual evaluation scores, for the dorsal and ventral regions of the ipsilateral and the contralateral hippocampus, respectively outlined in red and blue. Numbers indicate the total number of slices annotated with each score in each region.
